# Analysis of top influencers in critical care medicine “twitterverse” in the COVID-19 era: a descriptive study

**DOI:** 10.1186/s13054-021-03691-6

**Published:** 2021-07-20

**Authors:** Ronny Munoz-Acuna, Akiva Leibowitz, Margaret Hayes, Somnath Bose

**Affiliations:** 1 Department of Anesthesia, Critical Care and Pain Medicine, Beth Israel Deaconess Medical Center, Harvard Medical School, 330 Brookline Avenue, Boston, MA 02215 USA; 2 Division of Pulmonary, Critical Care, and Sleep Medicine, Beth Israel Deaconess Medical Center, Harvard Medical School, Boston, MA 02215 USA

**Keywords:** Intensive care medicine, Twitter, Social media, Internet, COVID-19, Network

Dear Editor:

Twitter, a microblogging platform, has become increasingly popular within the medical community as it facilitates prompt dissemination of information among users within and across specialties [1–3]. With a worldwide subscriber base of over 190 million, Twitter’s reach is broad and its impact substantial. Since the onset of the COVID-19 pandemic, the quantity of medical information shared through the platform has grown exponentially. Unfortunately, the veracity of the content disseminated is frequently unclear. Besides, the brevity of the information limits the ability to convey and interpret complex ideas, promoting valid and invalid *ad hominem* arguments as substantial forces in propagating ideas. Furthermore, misinformation spread through social media can lead to harm [4]. Therefore, it is essential to identify the main actors in the field since the top influencers are not necessarily experts in this area.

We aimed to characterize the demographics, academic credentials and research productivity of the top 250 critical care medicine influencers on Twitter as identified by proprietary software, Cronycle (London, UK) which uses a proprietary algorithm to calculate an influencer score based on engagement (which includes features such as retweets, likes and views) to determine the “influence” of a Twitter account within a topic of discussion [5]. This was performed on March 30, 2021, taking into consideration the following time period March 30, 2020–March 30, 2021, which coincided with the first wave of the ongoing pandemic. A network graph was created using NodeXL (Social Media Research Foundation, CA, USA) utilizing the last 1000 tweets of each account and establishing a visual relationship between the different accounts as shown in Fig. 1 [6]. To retrieve each influencer’s information, we looked at Twitter pages, Doximity accounts, LinkedIn profiles and institutional webpages. The h-index was obtained using the Scopus Preview Website.

**Fig. 1:**
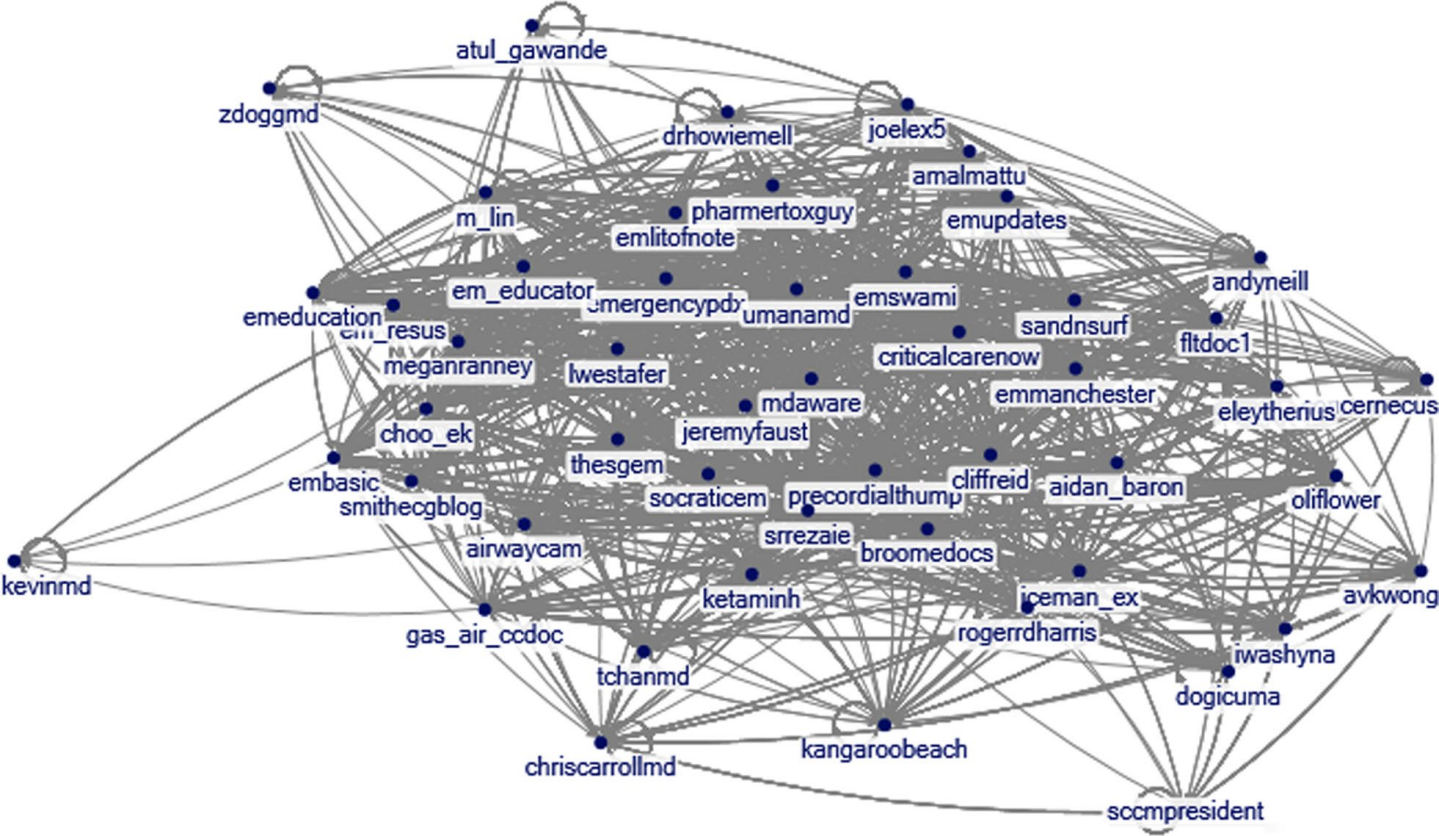
Relationship between the 50 top influencers in Critical Care Medicine on Twitter. Using NodeXL Pro the intricate relationship between the 50 most influential accounts of the CCM “twitterverse” is illustrated. Relationships shown are formed by using the last 1000 interactions (tweets, retweets, comments, shares and likes) of each individual account as of March 30, 2021

Among the top 50 influencers, only 28% (*n* = 14) had formal training in Critical Care Medicine (CCM). 84% (*n* = 42) of the top influencers were male. Emergency medicine was the most common specialty (*n* = 35, 70%), followed by anesthesia (*n* = 4, 8%), pulmonary critical care medicine (*n* = 2, 4%), internal medicine (*n* = 2, 4%), surgery (*n* = 1, 2%), pediatrics (*n* = 1, 2%). Most influencers (*n* = 31, 62%) held academic titles.

Further, we compared the demographic and academic credentials of the top 50 influencers with those in the lowest quintile (rank 201–250). The top influencers had a higher median influencer score 87 (IQR: 86, 89) vs. 75 (IQR: 74, 76) (*p* < 0.01) and higher number of followers 15,118 (IQR: 10,031, 27,543) vs. 3699 (IQR: 2443, 6688) (*p* < 0.01) when compared to the lowest quintile. Top influencers were more likely to work in academic settings (*p* = 0.006) when compared to those in the bottom 50. There was no statistically significant difference between h-indices (*p* = 0.902), number of publications (*p* = 0.935), number of citations (*p* = 0.946) or formal CCM training (*p* = 0.96) between the group of influencers.

Our descriptive study demonstrates that the CCM “twitterverse” is dominated by male US-based academic physicians mostly without fellowship training in CCM and modest scholarly productivity evidenced by publication, citations and h-indices (Table 1). Emergency medicine was the most predominant specialty represented among the top influencers. Limitations of using a proprietary software notwithstanding, our results indicate that the reach of influencers remains significant and is not correlated with academic productivity. Our limited sample does not allow us to draw overarching conclusions and should be considered exploratory; it is essential to consider that the veracity of the disseminated information may not necessarily correlate with the academic credentials or productivity and that the associations noted are highlighted solely for descriptive purposes. Further investigation in this area should focus on development of mechanisms categorizing tweets by their scientific content and validity.

**Table 1:** Characteristics of top 50 influencers and lower 50 influencers

Characteristic	Top 50 Influencers (Rank 1–50)	Lower 50 Influencers (Rank 201–250)	*P* value
	N (%)	N (%)	
Account characteristics			
Topic score	87 (IQR: 86–89)	75 (IQR: 74–76)	*p* < 0.01
Following	1082 (IQR: 315–2757)	1160 (IQR: 495–2163)	*p* = 0.694
Followers	15,118 (IQR: 10,031–27,543)	3699 (IQR: 2443–6688)	*p* < 0.01
Gender			*p* = 1.0
Male	42 (84%)	43 (86%)	
Academic indexes			
H-index	8 (IQR: 3–21)	8 (IQR: 3–16)	*p* = 0.902
Publications	26 (IQR: 6–72)	27 (IQR: 6–590)	*p* = 0.935
Citations	222 (IQR: 83–1910)	234 (IQR: 71–2666)	*p* = 0.946
Graduation year			
Medical school	2002 (IQR: 1995–2005)	2001 (IQR: 1998–2008)	*p* = 0.591
Residency	2005 (IQR: 2000–2011)	2004 (IQR: 2001–2011)	*p* = 0.725
Fellowship	2012 (IQR: 2008–2017)	2009 (IQR: 2002–2013)	*p* = 0.129
Location			*p* = 0.176
USA	30 (60%)	28 (56%)	
Other	20 (40%)	22 (44%)	
Practice setting			*p* = 0. 006
Academic	44 (88%)	38 (76%)	
Community	6 (12%)	8 (16%)	
Private		4 (8%)	
Faculty position			*p* = 0.445
Professor	13 (26%)	10 (20%)	
Associate professor	5 (10%)	6 (12%)	
Assistant professor	11 (22%)	4 (14%)	
Instructor	2 (4%)		
In-training	2 (4%)	3 (6%)	
CCM fellowship	14 (28%)	23 (46%)	*p* = 0.96

## Data Availability

Data will be made available upon reasonable request to researchers who provide a methodologically sound proposal, after approval by the study authors and with a signed data access agreement. Questions about data are handled by the corresponding author.

## References

[1] Valente TW, Pitts SR (2017). An appraisal of social network theory and analysis as applied to public health: challenges and opportunities. Annu Rev Public Health.

[2] Lee JL, DeCamp M, Dredze M, Chisolm MS, Berger ZD (2014). What are health-related users tweeting? A qualitative content analysis of health-related users and their messages on twitter. J Med Internet Res.

[3] Elfanagely Y, Atsawarungruangkit A, Moss SF (2021). Understanding GI Twitter and its major contributors. Gastroenterology.

[4] Rosenberg H, Syed S, Rezaie S (2020). The Twitter pandemic: The critical role of Twitter in the dissemination of medical information and misinformation during the COVID-19 pandemic. CJEM.

[5] Content Curation for Medical and Healthcare Communities|Cronycle Blog [Internet]. Cronycle. 2019 [cited 2021 Apr 2].

[6] Smith M, Ceni A, Milic-Frayling N, Shneiderman B, Mendes Rodrigues E, Leskovec J, Dunne C. NodeXL: a free and open network overview, discovery and exploration add-in for Excel 2007/2010/2013/2016, from the Social Media Research Foundation. 2010. https://www.smrfoundation.org.

